# Tumor-educated mesenchymal stem cells promote pro-metastatic phenotype

**DOI:** 10.18632/oncotarget.20265

**Published:** 2017-08-14

**Authors:** Billy Samuel Hill, Alessandra Pelagalli, Nunzia Passaro, Antonella Zannetti

**Affiliations:** ^1^ Institute of Biostructures and Bioimaging (IBB), National Research Council (CNR), Naples, Italy; ^2^ Department of Advanced Biomedical Sciences, University of Naples “Federico II”, Naples, Italy

**Keywords:** mesenchymal stem cells, tumor microenvironment, epithelial-mesenchymal transition, metastatic phenotype

## Abstract

Multipotent mesenchymal stem cells (MSCs) are recruited into tumor microenvironment in response to multiple signals produced by cancer cells. Molecules involved in their homing to tumors are the same inflammatory mediators produced by injured tissues: chemokines, cytokines and growth factors. When MSCs arrive into the tumor microenvironment these are “educated” to have pro-metastatic behaviour. Firstly, they promote cancer immunosuppression modulating both innate and adaptive immune systems. Moreover, tumor associated-MSCs trans-differentiating into cancer-associated fibroblasts can induce epithelial-mesenchymal-transition program in tumor cells. This process determinates a more aggressive phenotype of cancer cells by increasing their motility and invasiveness and favoring their dissemination to distant sites. In addition, MSCs are involved in the formation and modelling of pre-metastatic niches creating a supportive environment for colonization of circulating tumor cells.

The development of novel therapeutic approaches targeting the different functions of MSCs in promoting tumor progression as well as the mechanisms underlying their activities could enhance the efficacy of conventional and immune anti-cancer therapies.

Furthermore, many studies report the use of MSCs engineered to express different genes or as vehicle to specifically deliver novel drugs to tumors exploiting their strong tropism. Importantly, this approach can enhance local therapeutic efficacy and reduce the risk of systemic side effects.

## INTRODUCTION

In these last decades, many researches focused on the possible role of mesenchymal stem cells (MSCs) to promote tumor progression by interacting with tumor cells and other stroma cells in the complex network of microenvironment [1]. The multiple properties of these cells such as self-renewal, plasticity to differentiate into several cell types and ability to modulate immune response as well as strong tropism to tumors make them crucial players in the development of metastatic phenotype.

**Figure 1:**
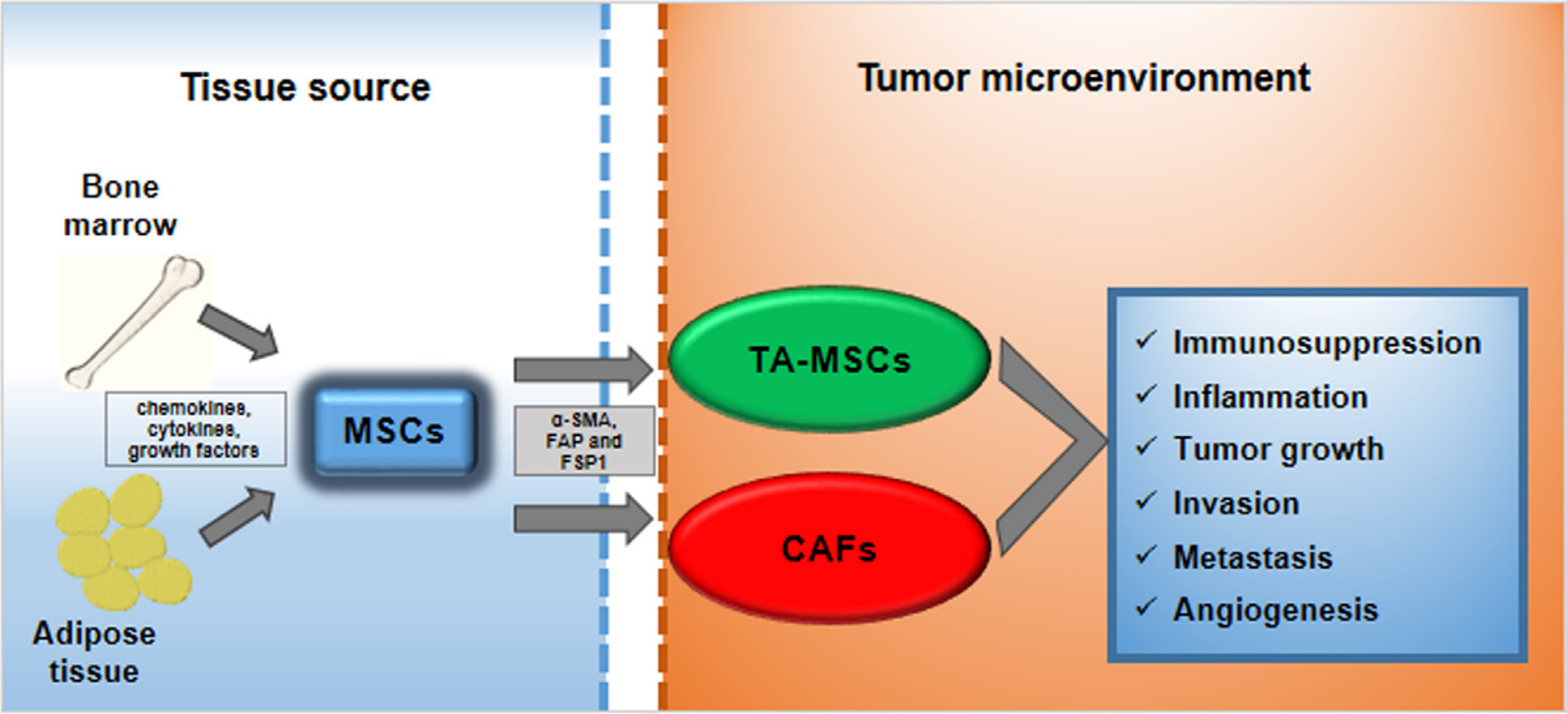
MSCs trans-differentiation into tumor microenvironment MSCs deriving from bone marrow or from adipose tissue can be recruited to tumor microenvironment in response to multiple signals: chemokines, cytokines and growth factors. Here, MSCs can trans-differentiate into tumor-associated MSCs (TA-MSCs) and cancer-associated fibroblasts (CAFs). They are characterized by specific markers such as α-smooth muscle actin (α-SMA), fibroblast activating protein (FAP) and fibroblast-specific protein 1 (FSP1). TA-MSCs and CAFs promote metastatic phenotype acting on: immunosuppression, inflammation, tumor growth, invasion, metastasis and angiogenesis.

Once the MSCs come into contact with the tumor microenvironment (TME) they are “educated” to evolve and differentiate in tumor-associated MSCs (TA-MSCs) and cancer associated fibroblasts (CAFs) [2–4]. Both these cells cooperate to support all hallmarks of cancer including sustaining proliferative signaling, evading growth suppressors, resisting cell death, enabling replicative immortality, inducing angiogenesis, and activating invasion and metastasis [5] (Figure 1).

Many signals are involved in the cross-talk between TA-MSCs and other component of tumor microenvironment. Several chemokines, cytokines, growth factors and others are produced by tumor cells to recruit MSCs from bone marrow and adipose tissue. In turn, TA-MSCs release the same molecules to repress immune surveillance, to induce epithelial-mesenchymal transition (EMT) program and to promote tumor cell migration and invasion. Recently, on this topic we reported that bone-marrow MSCs (BM-MSCs) derived from patients induced the metastatic phenotype of osteosarcoma and hepatocellular carcinoma through stromal derived factor 1 (SDF-1)-C-X-C-Chemokine receptor type 4 (CXCR4) axis and also through the aquaporin 1 (AQP1) membrane channel [6, 7]. Furthermore, it has been observed that MSCs can contribute to prepare the pre-metastatic sites by inducing a favorable microenvironment for the colonization of circulating tumor cells [1].

Although many studies reported the pro-tumorigenic activity of MSCs many others showed their tumor suppressive properties [8].

Here, we mainly focused on the progress made to elucidate the key mechanisms in which MSCs are involved to promote a pro-metastatic phenotype.

Nowadays, there are few reports regarding the possibility to target MSCs to hamper cancer progression. However, recently Ramos et al. (2017) reported that the inhibition of Histone deacetylases 8 in MSCs derived from myeloproliferative neoplasms selectively decreases their hematopoietic-supporting ability [9].

## TROPISM OF MESENCHYMAL STEM CELLS TOWARDS TUMORS

Currently, there has been a heightened focus on the homing abilities of MSCs into tumors and their role in promoting tumor progression [10, 11] but the specific mechanisms behind this are not yet well elucidated. MSCs that show a strong tropism to cancer, are derived from bone marrow [12, 13], adipose tissue [14] and also the umbilical cord [15].

Nakamizo et al. (2005) isolated human MSCs (hMSCs) from the bone marrow and followed their fate labeled them using fluorescent protein. Then, they injected labeled-MSCs into the opposite hemisphere of mouse brain of a orthotopic glioma model and after 14 days observed that the fluorescent hMSCs were exclusively within the brain tumors [13].

Moreover, fluorescent-labeled MSCs were detected in metastatic breast tumors in mice after their systemic administration and monocyte chemotactic protein-1 (MCP-1) produced by tumor cells was involved in their recruitment [16].

Interestingly, it has been reported that human adipose-derived MSCs (AD-MSCs) transduced with a retroviral vector encoding full-length human tumor necrosis factor-related apoptosis-inducing ligand (TRAIL) specifically localized into xenografts and mediated tumor cell apoptosis without significant apparent toxicities to normal tissues [14].

Furthermore, Hu et al. (2011) observed that human umbilical blood mononuclear cell (UBMC)-derived mesenchymal stem cells (UBMC-MSCs) transfected with recombinant pIRES2-IL-21-enhancement green fluorescent protein delayed tumor growth and prolonged survival in ovarian-cancer-bearing mice [15].

Tumors are considered the “wounds that never heal” and due to this are in a constant inflammatory status [17]. Indeed, cancer cells and tumor associated stroma cells produce various inflammatory molecules such as cytokines, chemokines and growth factors that are involved in MSC recruitment [18–20]. Moreover, it has been reported that MSCs respond to these signals by expressing high levels of specific receptors, adhesion molecules, cell surface markers and toll-like receptors [21].

At present, although much research is being carried out in this field, it has not yet been cleared which factor could play the most important role in this mechanism, it would seem that more factors are involved in the mediation of MSC tropism to a specific tumor site. In TME there is a chemotactic gradient consisting of different chemokines: CC-chemokine ligand 2 and 5 (CCL2, CCL5), CXC – chemokine ligand 12 and 16 (CXCL12, CXCL16), which are able to recruit MSCs [1]. Recently, it has been reported that the chemokine CCL2 plays a crucial role in the ionizing radiation induced tropism of MSCs to gliomas [22]. Furthermore, it has been shown that CCL2 and CCL5 produced by macrophages induce MSC recruitment in tumor sites [23]. Interestingly, a study evaluated the role of SDF-1a/CXCL12 in the migration of MSCs in response to tumor cells elucidating that Jak/STAT, MEK/ERK as well as NFkB pathways are activated downstream of SDF-1 [24]. Jung et al. (2013) provided evidence of an important cross-talk between MSCs and tumor cells in promoting a metastatic phenotype of prostate tumors. MSCs were both recruited as well as transformed in CAFs by CXCL16 produced by tumor cells. In turn, MSC-like CAFs secreted CXCL12 that binding to CXCR4 on tumor cells induced an EMT, which ultimately promoted metastasis to secondary tumor sites [25].

Many growth factors such as, platelet derived growth factor (PDGF), vascular endothelial growth factor (VEGF), insulin-like growth factor 1 (IGF-1), transforming growth factor-β (TGF-β) and basic fibroblast growth factor (bFGF) have been found to effectively mediate MSC homing to TME [21, 26, 27].

Doucette et al. (2011) showed that syngeneic MSCs are capable of homing to endogenous gliomas in immunocompetent mice. This model of high-grade glioma was induced by overexpression of PDGF-BB, that previously has been well characterized to be a critical mediator of MSC tropism to tumor sites [28–30].

Recent studies suggest that TGF-β1 is a crucial player in inducing MSC migration towards prostatic carcinoma cells (PC3 DU145) as well as to tumor stroma components [31]. In addition, Beckermann et al. (2008) showed that MSCs migrated towards growth factors produced by pancreatic tumors, such as PDGF, EGF, VEGF and that specific inhibitors Glivec, Erbitux and Avastin hampered their recruitment [32]. Furthermore, it has been demonstrated that bFGF and downstream Erk/Smad3 signaling pathway are involved in BM-MSC tropism to 4T1 breast cancer cells by using a specific neutralizing antibody [33].

In breast cancer, hypoxic TME, through activation of hypoxia-inducible factors (HIFs), is able to regulate the complex bidirectional MSC-tumor cell interaction. It has been demonstrated that hypoxia-induced expression of Placental growth factor (PGF) and CXCL16 in breast cancer cells is required for MSC recruitment and their pro-metastatic activity [33–36]. Likewise, breast cancer cells under hypoxic conditions (1.5% O_2_) were able to secrete high levels of interleukin-6 (IL-6), which served to activate and attract MSCs through Stat3 and MAPK signaling pathways [34].

MicroRNA's (miRNAs) have been shown to modulate MSC migration towards breast cancer. Indeed, miR-126/miR-126(*) suppressed the sequential recruitment of MSCs and inflammatory monocytes into the tumor stroma by directly inhibiting SDF-1α expression by cancer cells. [37].

A critical event of MSCs homing to cancer is to execute trans-migration through endothelial cells of the vessel. It has been observed that similarly to hematopoietic cells, MSCs express high levels of E-selectin, whereas lack the expression of platelet endothelial cell adhesion molecule-1 (PECAM-1), L-selectin and β2 integrins [38]. On the other hand, intravital microscopy demonstrated the capacity of MSCs to roll and adhere to post-capillary venules *in vivo* in a mouse model through a P-selectin and vascular cell adhesion molecule-1 (VCAM-1)/ very late antigen-4 (VLA-4) dependent manner [39].

At the present, many studies have been performed and are still underway to clarify the mechanisms underlying MSC tumor tropism and to evidence responsible factors which induce their recruitment in different tumor sites (Table 1).

**Table 1:** Factors involved in mesenchymal stem cell tropism to tumor microenvironment

	Factor	Tumor Type	Reference
**Cytokines**	TFN-a	Glioma	[151]
	IFN-g	Glioma	[151]
	IL-1b	Melanoma Breast Cancer	[152]
	IL-6	Breast Cancer	[34, 153]
	IL-8	Lung Cancer Breast Cancer	[154]
**Growth Factors**	TGF-b	Glioma Prostate Cancer	[31, 155]
	PGF	Breast Cancer	[35]
	PDGF	Glioma Renal Cancer Breast Cancer	[29, 30, 156]
	HGF	Gastric Cancer Lung Cancer	[157]
**Chemokines**	SDF-1/CXCR4	Glioma Breast Cancer Skin Cancer	[24, 154]
	CCL2 CCL5	Glioma	[22, 23]
	CXCL (GRO-a)	Breast Cancer	[153, 160]
	MCP-1	Breast Cancer	[16]
**Other Factors**	HIF-1	Glioma Breast Cancer	[35]
	MMP	Glioma	[158]
	VCAM	Glioma	[159]
	LL-37	Ovarian Cancer	[161]

## MESENCHYMAL STEM CELLS MODULATE TUMOR IMMUNE RESPONSE

MSCs are well known to be important modulators of inflammatory and immune responses affecting both the adaptive as well as innate immune systems [40]. In 1998, McIntosh KR et al. reported, for the first time, the use of MSCs for prevention and immune responses in transplantation [41]. A few years after, haploidentical MSCs were transplanted to treat severe acute graft-versus-host disease [42].

In TME cancer cells produce many inflammatory factors that are able to recruit non only MSCs but also many immune cells. A described above, MSCs into tumor sites are modified in TA-MSCs that contribute to generate malignant phenotype also excercising an immunosuppressive funcion [43, 44]. Han et al. (2011) reported that, when B16 melanoma cells were co-injected with MSCs pre-incubated with interferon-γ (IFN-γ) and tumor necrosis factor-α (TNF-α) in syngeneic mice, xenografts were developed faster than those obtained from B16 cells alone whereas tumor incidence was increased in allogeneic recipients [45]. These immunosuppressive effects were due to an increase of inducible nitric oxide synthase (iNOS) expression in MSCs [45]. Similarly, MSCs pre-treated with IL-1α effectively promoted the growth of prostate cancer cells *in vivo* through TGF-β upregulation [46].

In addition, It has been shown that MSCs isolated from spontaneous lymphomas in mice (L-MSCs) were more effective in recruiting monocytes/macrophages and in promoting tumor growth than BM-MSCs and their activity was mediated via C-C-Chemokine receptor type 2 (CCR2). Importantly, when BM-MSCs were TNFα-pretreated they mimicked L-MSCs in their chemokine production profile and in their ability to promote tumorigenesis not only of lymphoma but also melanoma, and breast carcinoma [47]. Recently, Yu et al. (2016) showed that TNFα-activated MSCs expressed CXCR2 ligands (CXCL1, CXCL 2 and CXCL5) and through them efficiently recruited CXCR2+ neutrophils into breast cancer microenvironment.

These neutrophils directly enhanced tumor lung metastasis, inducing tumor cells to express pro-metastatic genes [48]. In addition, in breast cancer cells indoleamine 2,3-dioxygenase (IDO)-expressing humanized MSCs (MSC-IDO) were capable of suppressing T-lymphocyte proliferation *in vitro* as well as reducing tumor-infiltrating CD8+ T cells and B cells *in vivo*, similar effects were also observed in melanoma and lymphoma tumor models [49].

Conversely, in breast cancer MSCs conferred immune protection through TGF-β1-mediated generation of forkhead box P3 (FoxP3)+ T_regs_ (regulatory t cells), that in turn suppressed tumor cell cytolysis by CD8+ T cells and natural killer (NK) cells [50]. In triple negative breast cancer, the cross-talk between tumor cells and MSCs, caused the production of macrophage colony-stimulating factor 1 (CSF1) that recruited in the TME, tumor-associated macrophages (TAMs) and myeloid-derived suppressor cells (MDSCs) [51]. Macrophages and MSCs may engage in a bidirectional interaction where M2 or M2-like macrophages determinate an increase of MSCs growth and motility [52]. In turn, MSCs can induce macrophages to acquire an anti-inflammatory phenotype with immunosuppressive abilities and pro-tumor functions [53, 54]. Recently, Yamada et al. (2016) reported that mouse BM-MSCs *in vivo* caused the increase of melanoma growth and M2 macrophage polarization through milk fat globule EGP factor 8 protein (MFG-E8) [55]. BM-MSCs obtained from patients with follicular lymphoma showed a different gene expression profile respect to MSCs obtained from healthy donors (HD-MSCs). These cells were able to recruit and polarize monocytes more efficiently than HD-MSCs thus sustaining malignant B-cell growth. Conversely, when MSCs were transfected to overexpress an NAD-dependent deacetylase sirtuin 1 (MSCs-Sirt1), they inhibited the growth of breast and prostate carcinomas by recruiting NK cells and macrophages [56]. Interestingly, MSCs associated in pancreatic carcinoma microenvironment had an increased tumor-promoting potential in respect to MSCs obtained from normal pancreas. This effect was mediated by their ability to induce macrophage polarization [57]. Chiassone et al (2016) showed that MSCs were able to induce the polarization of macrophages toward a novel M2-like phenotype (M^MSC^) that in turn could inhibit NK cells activation and could cause the expansion of T_regs_ cells [58]. In addition, the engagement of tool-like receptor (TLR) reverted M^MSC^ toward a M1 phenotype with pro-inflammatory and immunostimulatory activities [58] thus becoming detrimental for tumor progression. Conversely, it has been reported that MSCs derived from bone marrow of patients with low/intermediate risk leukemia at diagnosis enhanced the NK cell antitumor cytolytic activity and their pro-inflammatory cytokine production [59].

## TRANS-DIFFERENTIATION OF TUMOR-ASSOCIATED MESENCHYMAL STEM CELLS INTO CANCER ASSOCIATED FIBROBLASTS

When MSCs arrive into TME they can differentiate not only in TA-MSCs but also in CAFs. Among stromal cells that constitute TME, CAFs are known to play a crucial role in promoting tumor progression [60]. They are involved in all tumor events preceding the metastatic spread such as of EMT, neo-angiogenesis, immune surveillance, tumor cell migration and invasion [60]. CAFs were found in different forms of cancer and their high heterogeneity probably was due to different sources: fibroblasts, smooth muscle cells, endothelial cells and epithelial cells [61]. Recently, it has been reported that important CAF precursors are MSCs. These cells for their high plastic abilities, when stimulated directly or indirectly by factors produced from tumor cells or others stroma cells can trans-differentiate into a CAF-like phenotype [1]. For the first time, Mishra et al. (2008) observed that the effect of prolonged exposure of MSCs to factors produced by a human breast cancer cell line MDA-MB-231 caused up-regulation of 53 CAF-associated genes and an higher expression of α smooth muscle actin (α-SMA), vimentin, fibroblast surface protein (FSP) and SDF-1 [2]. An important evidence that CAF may derive from MSCs was shown by injecting green fluorescent protein (GFP)-labelled BM-MSCs in a mouse model of inflammation-dependent interleukin 1β (IL 1β) gastric cancer. About 20% of CAFs were found to be GFP-positive, indicating that GFP-BM-MSCs were their pre-cursors [4]. Importantly, in 2009 the first report was published showing that in female patients with gastric cancer and rectal adenoma, which had received bone marrow transplants from male donors, were identified several Y-chromosome positive CAFs [62].

In the last years, many studies investigated the key role played by TGF-β in the mechanism underlying MSC differentiation to CAFs. Recently, an interesting paper reported that in prostate cancer, MSCs are both recruited as well as induced to differentiate into CAFs in response to TGF-β produced by tumor cells. In addition, respect to normal MSCs, CAF-like MSCs performed vascular mimicry and recruited monocytes, which were polarized to M2 macrophages within the prostate cancer (PCa) environment [31]. Previously, Shangguan et al. (2012) observed that, when human BM-MSCs were transduced with a lentiviral vector encoding bone morphogenetic protein and activin membrane-bound inhibitor (BAMBI, a decoy TGF-β receptor), TGF-β/Smad signaling was significantly inhibited. Consequently, CAF markers were down-regulated in human BM-MSCs treated with TGF-β1 or tumor-conditioned medium or co-cultured with cancer cells [63]. Moreover, it has been reported that an endoplasmic reticulum (ER) chaperone GRP78, overexpressed in a variety of tumors, was able to induce BM-MSCs differentiation into CAFs through activating TGF-β/Smad signalling pathway. The importance of TGF-β involvement in MSC transformation was confirmed by another study where a TGF-β type I receptor kinase inhibitor, SB431542, caused a significant decrease of CAF markers expression [64].

It has also been observed that BM-MSCs were able to migrate towards 4T1 breast cancer cells and there trans-differentiate into CAFs in response to bFGF signaling pathway [33].

Several CAFs markers were identified in trans-differentiated MSCs, the main ones are α-SMA, the fibroblast activation protein (FAP) and FSP, but also thrombospondin-1, tenascin-C, desmin-1, and VEGF-AA can be involved [5, 65, 66]. α-SMA has been known to play a pivotal role in the embryonic stem cell-derived cardiomyocyte differentiation. On the other hand, expression of α-SMA in the stroma increases fibroblasts contractile ability and contributes to alterations in the cytoskeletal organization [61, 66, 67]. CAFs may alter the extracellular matrix (ECM) through the production of proteases such as FAP. High levels of this enzyme are expressed in over 90% of human epithelial carcinomas including breast, lung, and ovarian cancers. Conversely, normal healthy adult tissues have almost no detectable FAP expression [68]. Generally, MSC-like CAFs that are positive for FSP and FAP originate from MSCs which derive from the bone marrow, whereas it has been observed that adipose-derived MSC mainly differentiate into vascular and fibrovascular stromal cells. [65]. Compared to normal fibroblasts and myofibroblasts, CAFs are perpetually activated, and cannot revert back to their original phenotype nor undergo apoptosis [61], confirming their dramatic role in tumor progression.

Recently, some reports have suggested that the irreversible activation of fibroblasts might be driven by epigenetic alterations [69, 70]. Albrengues et al (2015) identified the DNA methyltransferase (DNMT) family epigenetic modifiers as regulators of the pro-invasive CAF activity that affects Janus kinase 1 (JAK1)–signal transducer and activator of transcription 3 (STAT3) activation [71]. Dual inhibition of DNMT and JAK activity restored the non-invasive phenotype of CAFs. Conversely, a global gene hypometylation was observed in CAFs isolated from human gastric cancers [72]. Moreover, Shakya et al found globally decreased 5-methyl-cytosine (5-mC), along with increased amounts of 5-hydroxymethyl-cytosine (5-HmC) in CAFs, in progression from pancreatic intraepithelial neoplasia to pancreatic ductal adenocarcinoma [73]. Among these epigenic changes the post-trascriptional control involving miRNA statutes plays an important role [69]. For example, it has been observed that upregulation of miRNA-21 in CAFs is associated with high proliferation in breast cancer [74, 75], poor-disease-free survival in colorectal carcinoma [76] and invasion of esophageal squamous cell carcinoma [77].

Notably, CAFs are able also to migrate with epithelial cancer cells through endothelial cell layers, thus contributing to establishment of new pre-metastatic niches [78–80].

## MESENCHYMAL STEM CELLS PROMOTE CANCER CELL EPITHELIAL–MESENCHYMAL TRANSITION AND INVASION

**Figure 2:**
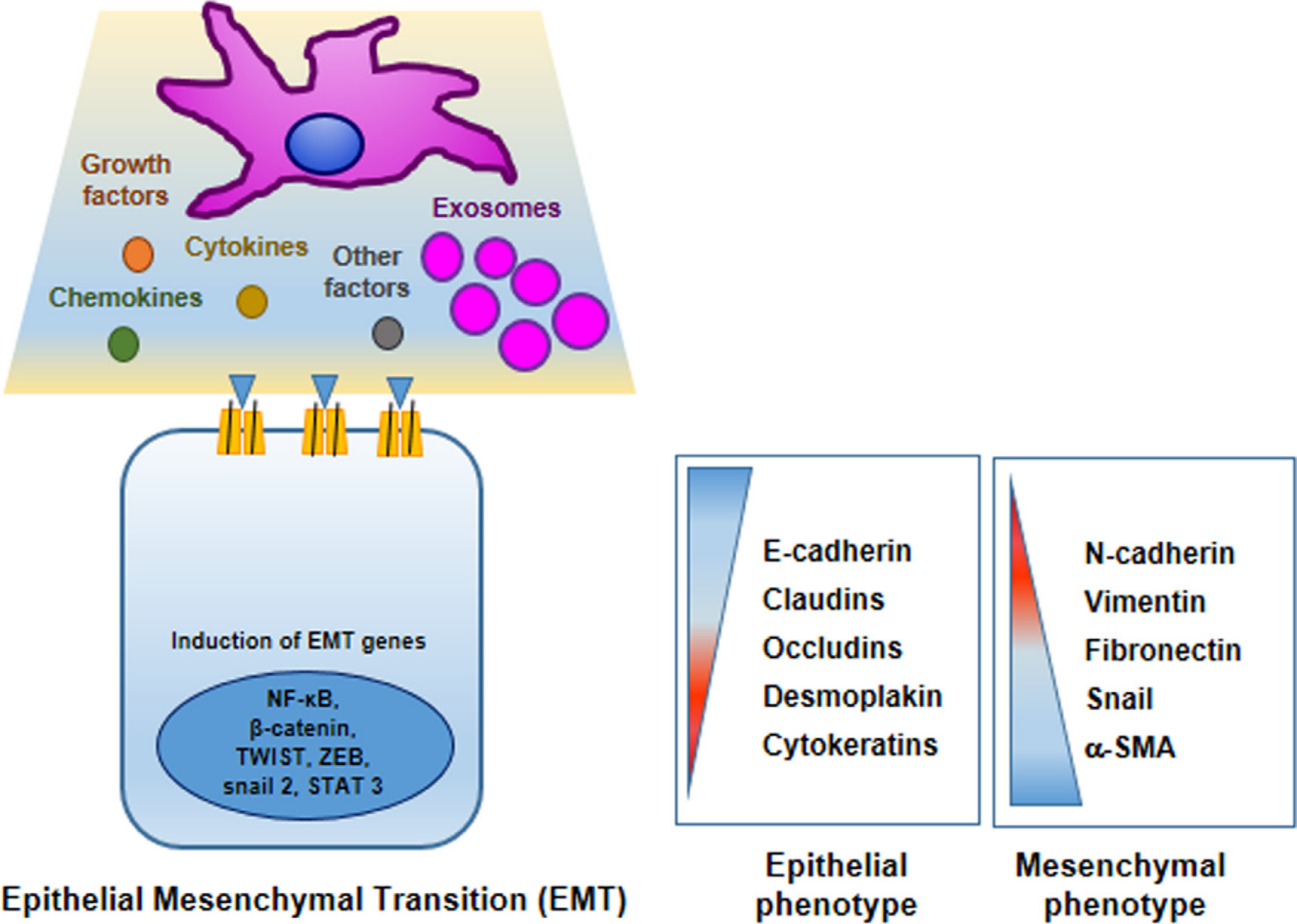
Mesenchymal stem cells promote cancer cell epithelial–mesenchymal transition (EMT) MSCs in TME produce many factors that induce or repress in tumor cells different genes encoding proteins involved in EMT program. In tumor cells epithelial markers are down-regulated: E-cadherin, claudins, occludins, desmoplakin and cytokeratins; whereas mesenchymal markers are up-regulated: N-cadherin, vimentin, fibronectin, snail and and smooth muscle actin.

In these last years, research studies on mechanisms underlying tumor progression and acquisition of pre-metastatic phenotype have demonstrated well that MSCs actively participate in inducing oncogenic EMT (Figure 2) [81, 82].

In particular, in TME where a complex crosstalk between tumor cells and stromal cells takes place, the epithelial tumor cells undergo profound morphological and functional changes trans-differentiating in a mesenchymal-like phenotype with a higher metastatic potential [83]. Generally, EMT represents a physiological developmental process by which the epithelial cells in particular conditions (normal embryogenesis, tissue repair and in tumors), upon extracellular cues, undergo profound morphogenetic changes, to become cells with phenotype and morphology similar to stem cells [81, 84]. EMT of tumor cells is characterized by a sequence of molecular events: 1) down-regulation of E-cadherin, 2) secretion of enzymes (i.e. matrix metalloproteinases), 3) up regulation of mesenchymal markers (i.e vimentin, N-cadherin and fibronectin) [85, 86].

Many EMT inducible factors have been demonstrated to be produced or secreted by MSCs such as cytokines (IL1, IL6) [87], chemokines (CCL5, CXCL1, CXCL5, CXCL7 and CXCL8) [88, 89], growth factors (TGF-β, FGF, *Hepatocyte growth factor* HGF and epidermal growth factor, EGF) [90, 91] as well as hypoxia inducible factors and reactive oxygen species. Interestengly, in breast cancer a part from MSCs, other stromal cells such as adipocytes are able to promote a more aggressive phenotype by producing CCL5 and IGF-1 [92, 93].

These molecules acting in a paracrine manner are able to orchestrate EMT program. It has been reported the importance of expression and functional activation of a group of EMT-inducing transcription factors (Twist, Snail, Slug, zinc finger E-box-binding homeobox 1 (ZEB1) and ZEB2 [81, 94]. The activation of these transcription factors causes the down-regulation of genes responsible to encode epithelial junction proteins determining a disassembly of adherens junctions, desmosomes, and tight junctions [95]. Conversely, they can up-regulate expression of mesenchymal genes encoding N-cadherin, fibronectin and vimentin [96]. The trigger of these transcription factors is sufficient to induce EMT in tumor epithelial cells as observed in different invasive carcinomas [97]. On this regard, recently we reported that CXCL12/CXCR4 axis is involved in migration and invasion of osteosarcoma and hepatocellular carcinoma cell lines through EMT activation [6].

The pivotal role of EMT program in cancer progression, has been investigated in several pre-clinical cancer models such as breast [98], ovarian [99], colon [100], and esophageal carcinomas [101].

Most recently, it has been reported that EMT program is also under miRNAs control that may promote mesenchymal or inhibit epithelial gene expression. miRNA-9 which is up-regulated in breast cancer cells, directly targets the E-cadherin-encoding messenger RNA, leading to an increase in cell motility and invasiveness [102]. Conversely, miRNA 200b was able to revert EMT in prostate carcinoma animal model increasing pan-epithelial marker such as E-cadherin, cytokeratins 8 and 1 and down-regulating mesenchymal markers, fibronectin and vimentin [103]. Furthermore, miRNA-21 promoted the acquisition of luminal markers and EMT in prostate cells suppressing B-cell translocation gene 2 (BTG2) expression [104].

In conclusion, EMT process can be drawn as very interesting and complex phenomena that offers a way to move cancer cells from primary tumor to the confined tissues and blood vessels thus facilitating metastatization process [85]. In addition, it has been well demonstrated that tumor-like mesenchymal stem cell cells can also undergo a reverse phenotype switching to again become phenotypically epithelial cells via mesenchymal-to-epithelial transition (MET) [105]. This process is associated with formation of metastatic niches [97].

A particular pre-requisite for tumor invasion is represented by modifications of ECM involving the degradation of its components by enzymes as the metalloproteinases 2 and 9 (MMP-2 and MMP-9) [106–108]. It has been reported that MSCs increased MMPs expression in lung and pancreas carcinoma activating EMT program [109, 110]. In particular, it seem plausible that MMPs could be directly involved in EMT program during cancer progression by different mechanisms: (a) the increase of MMPs in the tumor microenvironment can prompt EMT in epithelial cells, (b) cancer cells that use EMT programming produce extra MMPs and (c) epithelial cells can be subjected to EMT through additional MMP [111]. MSCs can also act on ECM modifying its organization by production of molecules as collagen [112]. Furthermore, Kaplan et al. (2005) investigating the role of MSCs to contribute to pre-metastatic sites, observed that bone marrow-derived hematopoietic progenitor cells that expressed vascular endothelial growth factor receptor 1 (VEGFR1; also known as Flt1) homed to tumor-specific pre-metastatic sites and formed cellular clusters before the arrival of tumor cells [113].

## MESENCHYMAL STEM CELLS AS VEHICLES FOR CELL-BASED CANCER THERAPY

MSCs are being investigated as cellular vehicles for the delivery of anti-cancer agents on the basis of their pronounced tropism and integration capacity into tumor microenvironment as well as for their immunomodulatory abilities [114] (Table 2). Indeed, MSCs can be infused into HLA-non-identical recipients because they do not activate the host immune response and escape immunological rejection [115]. They may be genetically engineered to express anti-proliferative, anti-angiogenic, pro-apoptotic factors as well as cytokines and suicide genes. Recently, Marini et al. reported the therapeutic activity of MSC stably transfected to express a TNF-Related Apoptosis-Inducing Ligand (TRAIL)-EGFR specific against Colo205 xenograft tumor model [116]. Similarly, when MSC expressing TRAIL were injected in pre-established Ewing's Sarcoma mouse model they caused significant tumor apoptosis and showed anti-angiogenic function respect to control group [117]. It has been observed that MSCs engineered to produce and deliver scFv-Ftd-tBid, a novel γ-SM-targetd immuno-proapoptotic molecule, inhibited prostate cancer growth both *in vitro* and *in vivo* [118]. Furthermore, MSCs combined with an adenovirus vector to deliver NK4 caused a decrease of growth and migration of high metastatic liver carcinoma cells as well as neo-angiogenesis [119]. Many other anti-angiogenic approaches have been used to impair tumor progression using MSCs as cargo. These cells have been engineered to produce anti-angiogenic factors in different tumor models such as soluble VEGF receptor-1 (sFLT-1) in Lewis lung cancer [120], endostatin in colorectal carcinoma [121], throspondin-1 (TSP-1) in glioblastoma [122] and pigment epithelium-derived factor (PEDF) in prostate carinoma [123]. Interestingly, MSCs can be transfected to express different cytokines to elicit an immune response and/or to inhibit tumor progression. Several findings reported the ability of IFN-β gene modified MSCs to reduce tumor growth in hepatocellular carcinoma [124], glioblastoma [125] and pancreatic tumors [126]. In addition, it has been observed that the secretion of IL-12 from engineered MSCs in tumor microenvironment of different cancer models such as glioma, Ewing sarcoma, renal cell carcinoma and breast cancer, causes reduction of tumor growth [126–130]. Another interesting therapeutic approach use MSCs loaded with suicide genes such as the herpes simplex virus-thimidine kinase (HSV-TK) and cytosine deaminase (CD) to hamper tumor progression. HSV-TK converts non-toxic ganciclovir (GCV) into phosphorylated toxic compound (GCV-ppp) whereas CD modifides low-toxic substrat 5-fluorocytosine into a 5-fluorouracil potent anticancer agent, in both cases MSCs delivery efficiently these genes in tumor sites. A significant decrease in tumor growth and a subsequent increase in survival were observed when mice bearing highly aggressive GBM were treated with MSC co-expressing S-TRAIL and HSV-TK [131], while sequential combination gene therapy using MSC/dTRAIL-TK achieved long-term remission of metastatic renal cell carcinoma without noticeable toxicity [132]. Importantly, it has been reported a single-arm phase I/II study which assessed the safety and efficacy of HSV-TK genetically modified autologous MSCs as delivery vehicles for a cell-based gene therapy, for advanced recurrent, metastatic gastrointestinal and hepatopancreatobiliary adenocarcinoma [133]. Furtheremore, CD-MSCs showed high therapeutical efficacy to treat animal models of osteosarcoma [134], glioma [135], melanoma [136] and prostate carcinoma [137].

**Table 2:** Mesenchymal stem cell as cargo for anti-cancer agents

	Agent	Tumor Type	Reference
**Suicide genes**	HSV-TK	Glioma	[131]
		Renal Cell Carcinoma	[132]
		Gastrointestinal and Hepatopancreatobiliary adenocarcinoma	[133]
	CD	Osteosarcoma	[134]
		Glioma	[135]
		Melanoma	[136]
		Prostate carcinoma	[137]
**Anti-angiogenic factors**	Soluble VEGF Receptor-1	Lewis lung cancer	[120]
	Endostatin	Colorectal carcinoma	[121]
	Throspondin-1	Glioblastoma	[122]
	Interferon-b	Hepatocellular carcinoma	[124]
		Glioblastoma	[125]
		Pancreatic cancer	[126]
		Glioma	[127]
**Cytokines**	Interleukin-12	Ewing sarcoma	[128]
		Renal cell carcinoma	[129]
		Breast cancer	[130]
	Interleukin-18	Glioma	[162]
	Interleukin-21	Ovarian cancer	[121]
	Interleukin-15	Pancreatic cancer	[163]

HSV-TK: herpes simplex virus-thimidine kinase; CD: cytosine deaminase.

## ANTI-TUMORIGENIC ACTIVITY OF MESENCHYMAL STEM CELLS

MSCs derived from different sources and acting in different tumor contexts can have a pro- or an anti-tumorigenic behavior. Probably, these opposite effects of MSCs can depend on the experimental setting in cell and in the animal models. It seems that among the MSCs which that originate from umbilical cord /cord blood (UC-MSCs) show more anti-cancer properties. Many studies report that UC-MSCs not only suppress tumor progression, but also promote drug sensitivity of different blood cancer cells including Jurkat leukemia cells [138], K562 erythromyeloblastoid leukemia cells [139], Burkitt's limphoma cells [140] and multiple myeloma cells [141]. In addition, UC-MSCs exert a suppressive effect on tumor growth of several solid tumor cell lines: breast cancer [142], liver cancer [143], prostate and bladder cancer [144]. Similarly, adipose tissue MSCs (AT-MSCs) inhibit proliferation and induce apoptosis of hepatc cancer cells [145], breast cancer cells [146] as well as prostate cancer cells [147] and melanoma [148]. BM-MSCs mainly show pro-tumorigenic effects even if in some cases it has observed a their anti-proliferative activity on tumor growth [149, 150]. This peculiar aspect of MSCs to repress progression of specific tumors in particular conditions through production of endogenous factors suggest the possibility to use naïve MSCs not only as drug cargo but also themselves as anti-cancer agents.

## CONCLUSIONS AND PERSPECTIVES

**Figure 3:**
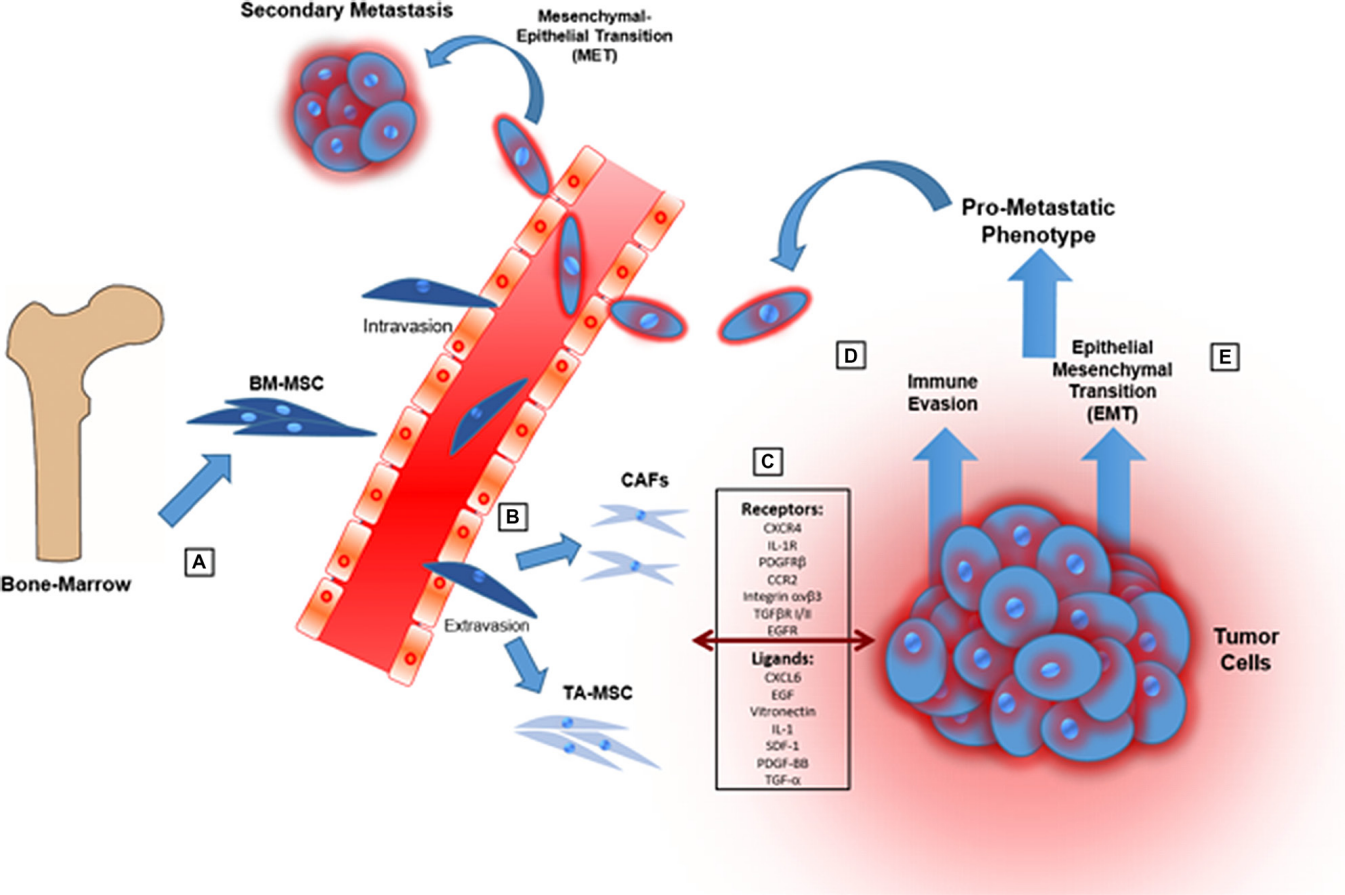
Cartoon showing the main steps through which MSCs promote tumor progression and a pro-metastatic phenotype (**A**) MSCs are recruited from bone-marrow and adipose tissue in response of mediators produced by cancer and stroma cells in TME. (**B**) MSCs into TME can be “educated” to evolve in tumor-associated MSCs (TA-MSCs) and differentiate in cancer-associated fibroblasts (CAFs). (**C**) MSCs can engage in bidirectional communication with tumor cells through different signals. (**D**) MSCs can promote immunosuppression in TME modulating both innate and adaptive immune systems. (**E**) MSCs induce epithelial-mesenchymal transition causing a more aggressive phenotype of tumor cells.

In the present review, we highlight the multiple activities of TA-MSCs in the TME with particular emphasis on their ability to stimulate cancer immunosuppression, to induce EMT program, to trans-differentiate into CAFs and finally to promote pro-metastatic phenotype (Figure 3).

Many mechanisms underlying MSCs role in TME have been elucidated but still further studies are needed to understand completely their involvement in tumor progression.

These investigations could provide information for targeting TA-MSCs with therapeutic approaches and eventually for using them as vehicles to deliver specifically in TME anti-cancer agents.
